# Effect of Obesity and Metabolic Health Status on Metabolic-Associated Steatotic Liver Disease among Renal Transplant Recipients Using Hepatic Steatosis Index

**DOI:** 10.3390/nu16193344

**Published:** 2024-10-01

**Authors:** I-Hsin Lin, Yi-Ping Yu, Tuyen Van Duong, Shih-Wei Nien, I-Hsin Tseng, Yi-Ming Wu, Yang-Jen Chiang, Chia-Yu Chiang, Chia-Hui Chiu, Ming-Hsu Wang, Nien-Chieh Yang, Ta-Ho Wu, Te-Chih Wong

**Affiliations:** 1 Department of Medical Nutrition Therapy, Linkou Chang Gung Memorial Hospital, Taoyuan 333, Taiwan; cabbage@cgmh.org.tw (I.-H.L.); nina0904@cgmh.org.tw (S.-W.N.); cathy40422@cgmh.org.tw (I.-H.T.); yiming1023@cgmh.org.tw (Y.-M.W.); 2 Department of Nutrition and Health Sciences, Chinese Culture University, Taipei 111, Taiwan; yyp@ulive.pccu.edu.tw (Y.-P.Y.); a9221185@ulive.pccu.edu.tw (N.-C.Y.); wdhwmb001@gmail.com (T.-H.W.); 3 School of Nutrition and Health Sciences, College of Nutrition, Taipei Medical University, Taipei 110, Taiwan; tvduong@tmu.edu.tw; 4 Department of Urology, Linkou Chang Gung Memorial Hospital, Taoyuan 333, Taiwan; zorro@cgmh.org.tw; 5 Department of Medicine, Chang Gung University, Taoyuan 333, Taiwan; 6 Department of Business Administration, College of Management, National Changhua University of Education, Changhua 500, Taiwan; cychiang@cc.ncue.edu.tw; 7 Center for General Education, Taipei Medical University, Taipei 110, Taiwan; thera@tmu.edu.tw (C.-H.C.); mattwang@tmu.edu.tw (M.-H.W.)

**Keywords:** renal transplant recipients, metabolically healthy obese, metabolic-associated fatty liver disease

## Abstract

Background/Objectives: Obesity and metabolic conditions increase the risk of metabolic-associated steatotic liver disease (MASLD). This study examined the risk of MASLD in 137 renal transplant recipients (RTRs) from a single-center hospital on the basis of their obesity and metabolic health status. Methods: Participants were categorized into four groups: metabolically healthy nonobese (MHNO), metabolically healthy obese (MHO), metabolically abnormal nonobese (MANO), and metabolically abnormal obese (MAO). MASLD was assessed using the hepatic steatosis index (HSI), calculated as 8 × (aspartate aminotransferase/alanine aminotransferase ratio) + body mass index + 2 (if diabetic) + 2 (if woman). The HSI scores were 29.50 ± 4.55, 38.08 ± 5.44, 33.61 ± 5.23, and 39.86 ± 4.13 in the MHNO, MHO, MANO, and MAO groups, respectively (*p* < 0.05). Results: Overall, 25.55% of the participants (57.14% men) were classified as having MASLD (HSI > 36). A multivariate-adjusted regression analysis revealed significantly higher HSI scores in the MAO group than in the MHNO group. Both MHO and MANO groups also had significantly higher HSI scores. The odds ratios for more severe MASLD were 2.74 (95% CI: 0.88–8.52) for the MANO group and 74.59 (95% CI: 13.29–418.68) for the MAO group compared with the MHNO group. Conclusions: These findings suggest that RTRs with obesity have a higher risk of MASLD, but even those with a normal weight and metabolic abnormalities are at increased risk.

## 1. Introduction

Metabolic dysfunction-associated steatotic liver disease (MASLD) is highly prevalent among renal transplant recipients (RTRs). In these patients, the use of immunosuppressive medications, such as steroids and calcineurin inhibitors, substantially increases the risk of metabolic complications [1]. Previous studies with smaller sample sizes have reported that the prevalence of MASLD is 42.3% [2]. Some studies have indicated a prevalence of more than 50% [3] among RTRs. However, related data from Asia are lacking.

Obesity, as measured by the body mass index (BMI) and metabolic risk factors, are indicated as key components in the proposed diagnostic criteria for MASLD [4]. Individuals with obesity who exhibit few or no metabolic abnormalities are classified as metabolically healthy obese (MHO). By contrast, individuals who are not obese but have metabolic abnormalities are categorized as metabolically abnormal nonobese (MANO) [5]. Although the relationships of obesity and metabolic health status with cardiovascular disease [6] and type 2 diabetes [7] have been widely studied, and these factors significantly contribute to the susceptibility of RTRs to MASLD, the specific roles of obesity and metabolic health in the development of MASLD, particularly among RTRs, remain unclear.

The aim of this study is to investigate the association of obesity and metabolic health status with MASLD in RTRs. We assessed MASLD by using the hepatic steatosis index (HSI), a simple and efficient screening tool [8], through an observational, single-center study design.

## 2. Participants and Methods

### 2.1. Study Design

This cross-sectional study included adult RTRs aged 20 years and above who had been receiving regular follow-up care at a district teaching hospital in northern Taiwan since September 2016. The need for written informed consent from patients was required for this study. Data were collected during participants’ follow-up visits while they were in a fasting state. Trained staff collected information on background characteristics, anthropometric measurements, laboratory tests, and dietary data, adhering to standardized procedures as previously detailed [9,10].

### 2.2. Study Population

Stable RTRs who were on a consistent immunosuppressive regimen, comprising calcineurin inhibitors (CNIs), antimetabolites, and steroids, were included. We excluded participants who had experienced acute rejection, had a body weight change of more than 3 kg, or had a glomerular filtration rate fluctuation of more than 25% within the past 3 months. In addition, patients were excluded if they had undergone more than two kidney transplants, received other organ transplants, had cancer, had substantial edema, were pregnant, had undergone amputations, or had hyperthyroidism or hypothyroidism, because these factors could affect the accuracy of the study findings. Patients who did not give their consent to participate or who had extreme dietary records (caloric intake below 800 or above 3000 calories per day) or incomplete data were also excluded. Finally, 137 participants were included in the analysis.

### 2.3. Background Characteristics and Anthropometric Measurements

Data on background characteristics, including medical history and medication use, were obtained from patients’ medical records and verified through face-to-face interviews during their follow-up visit. Waist circumference (WC) was measured at the narrowest point between the last rib and the iliac crest by using a tape measure. BMI was calculated by dividing each participant’s weight (in kilograms) by their height (in meters squared). Obesity was defined as a BMI of 27 kg/m^2^ or higher in accordance with guidelines from Taiwan’s Ministry of Health and Welfare [11]. Comorbidities were evaluated using the Charlson comorbidity index (CCI) [12], and data were collected from baseline surveys and chart reviews.

### 2.4. Laboratory Measurements

After an 8 h fasting period, venous blood samples were collected at the clinical laboratory of Chang Gung Memorial Hospital for biochemical analysis. The tests included assessments of albumin, blood urea nitrogen, creatinine, fasting blood glucose (FBS), glycated hemoglobin, uric acid, triglycerides, total cholesterol, low-density lipoprotein cholesterol, high-density lipoprotein cholesterol (HDL-C), aspartate aminotransferase (AST), alanine aminotransferase (ALT), and high-sensitivity C-reactive protein (hs-CRP). All analyses were performed using an automated analyzer (Sysmex XN-3000, Kobe, Japan) following standardized protocols. Insulin resistance was evaluated using the homeostatic model assessment for insulin resistance (HOMA-IR), calculated as HOMA-IR = (glucose in mg/dL) × (insulin in mU/L)/405 [13]. The estimated glomerular filtration rate (eGFR) was calculated using the modification of diet in renal disease (MDRD) formula [14]:

eGFR (mL/min/1.73 m^2^) = 175 × (serum creatinine)^−1.154^ × (Age)^−0.203^ × 0.742 (if woman)

### 2.5. Definition of Metabolic Health and Obesity Status

Participants were classified as metabolically unhealthy if they met at least three of the five following criteria [15]: (1) WC ≥ 90 cm for men and ≥80 cm for women, (2) FBS ≥ 100 mg/dL or current use of antidiabetic medications, (3) blood pressure ≥ 130/85 mmHg or current use of antihypertensive drugs, (4) triglyceride level ≥ 150 mg/dL or current use of lipid-lowering drugs, or (5) HDL-C < 40 mg/dL in men or <50 mg/dL in women. Participants who did not meet these criteria were considered metabolically healthy. Obesity was defined as a BMI of 27 kg/m^2^ or higher in accordance with Taiwan’s Ministry of Health and Welfare guidelines [11]. On the basis of these criteria, participants were divided into four groups: metabolically healthy nonobese (MHNO), metabolically healthy obese (MHO), MANO, and metabolically abnormal obese (MAO).

### 2.6. HSI

The HSI was calculated using the following formula: 8 × ALT/AST + BMI + 2 (if diabetic) + 2 (if woman) [8]. Diabetes was defined as an FBS level of ≥126 mg/dL, a prior diagnosis of diabetes by a physician, or the current use of antidiabetic medication. On the basis of a previous study, an HSI score greater than 36 was used as the threshold for identifying potential MASLD in the present study [16].

### 2.7. Statistical Analysis

Data were analyzed using SAS software, version 9.4 (SAS Institute, Cary, NC, USA). Normality was evaluated using the Shapiro–Wilk test and Q–Q plots. Student’s *t*-test was used to compare the means of normally distributed variables, whereas the Wilcoxon rank sum test was used for non-normally distributed variables. To summarize the characteristics of study participants on the basis of their metabolic health status, one-way analysis of variance and chi-square tests were performed where appropriate. Post hoc analyses were conducted using Scheffe’s adjustment to compare variables across different metabolic health and obesity groups. The association of the HSI with metabolic health and obesity status was examined using both linear and logistic regression analyses. Three models were developed to calculate multivariable-adjusted regression coefficients (β) and odds ratios (OR). The MHNO group served as the reference. The models included (1) a crude model; (2) Model 1, with adjustments for sex and age; and (3) Model 2, with adjustments for total energy intake, comorbidities (as indicated by the CCI), kidney transplant date, donor source, and immunosuppressant use. The variables included in each model were those known to affect the HSI or recognized as crucial prognostic factors. A *p* value of less than 0.05 was considered statistically significant.

## 3. Results

### 3.1. Characteristics of Participants

Table 1 summarizes the characteristics of the study participants. The mean age of the 137 participants who met the inclusion criteria was 49.20 ± 12.46 years, and 58.39% of them were men. The average time since transplantation was 8.34 ± 7.01 years. The mean blood urea nitrogen and creatinine levels were 24.07 ± 10.34 and 1.43 ± 0.70 mg/dL, respectively. The average eGFR was 55.48 ± 20.40 mL/min/1.73 m^2^, indicative of stage 3 chronic kidney disease.

Overall, 25.55% of participants (35 individuals, 57.14% men) were classified as having MASLD on the basis of their HSI (>36). Participants with an HSI of >36 had significantly higher values for obesity-related parameters, including body weight, BMI, and WC, as well as elevated levels of FBG, HbA1c, TG, ALT, insulin resistance, and hs-CRP. However, participants with an HSI of >36 had a significantly lower albumin levels than those with an HSI of <36.

### 3.2. Characteristics of Participants According to Obesity Phenotypes

Table 2 presents the characteristics of the study participants, categorized by metabolic health and obesity status. The prevalence rates of MHNO, MHO, MANO, and MAO were 56.20% (*n* = 77), 8.03% (*n* = 11), 24.09% (*n* = 33), and 11.68% (*n* = 16), respectively. Significant differences were observed among these groups in body weight; BMI; WC; systolic blood pressure; and albumin, FBG, HbA1c, TG, HDL-C, AST, ALT, insulin, insulin resistance, and hs-CRP levels.

The prevalence rates of MASLD, indicated by an HSI of >36, were 9.09% (*n* = 7), 54.55% (*n* = 6), 24.24% (*n* = 8), and 87.5% (*n* = 14) in the MHNO, MHO, MANO, and MAO groups, respectively (*p* < 0.0001). The HSI scores were 29.50 ± 4.55, 38.08 ± 5.44, 33.61 ± 5.23, and 39.86 ± 4.13 in the MHNO, MHO, MANO, and MAO groups, respectively (*p* < 0.05). Compared with the RTRs without obesity, those with obesity, regardless of their metabolic health, had significantly higher HSI scores.

### 3.3. Association between MASLD and Obesity Phenotypes

Table 3 presents the association between different obesity phenotypes and the HSI. A significant increase in the risk of MASLD was observed across all phenotypes. As expected, the MAO group had a significantly higher HSI than the MHNO group, independent of age, sex, transplant-related characteristics, and obesity-related parameters. Furthermore, the MHO and MANO groups had significantly higher HSI than the MHNO group, independent of age and sex. The regression coefficients (95% CIs) were 8.43 (4.71–12.15; *p* < 0.0001) for the MHO group and 3.88 (1.45–6.31; *p* = 0.0002) for the MANO group. Even after adjustment for transplant-related characteristics and obesity-related parameters, these associations remained significant: 8.01 (4.11–11.91; *p* < 0.0001) for the MHO group and 4.38 (1.81–6.94; *p* < 0.0001) for the MANO group.

Table 4 presents multiple logistic regression results on the likelihood of MASLD, defined by a HSI score of >36, for the MHO, MANO, and MAO groups and the MHNO group for comparison. Compared with that in the MHNO group, the risk of MASLD was significantly higher in the MANO and MAO groups, with ORs of 2.61 (0.86–7.88) and 66.34 (12.69–346.75), respectively, independent of age and sex; the difference between the MHNO and MHO groups was nonsignificant. After adjustment for covariates, including transplant-related characteristics and obesity-related parameters, the risk remained significantly higher in the MAO (OR, 74.59; 95% CI, 13.29–418.68) group but borderline significantly higher in the MANO group (OR, 2.74; 95% CI, 0.88–8.52) than in the MHNO group.

## 4. Discussion

To the best of our knowledge, this study is the first to investigate the combined effect of obesity and metabolic health on liver health in RTRs. Our findings revealed that both metabolic abnormalities and obesity significantly increase the risk of MASLD, as assessed using the HSI. In addition, even after adjustment for confounding factors, the MANO and MAO groups were more likely to develop MASLD, regardless of their obesity status. These results indicate the strong association among obesity, metabolic dysfunction, and liver health in RTRs, suggesting that routine post-transplant care should include comprehensive evaluations of both BMI and metabolic risk factors.

In the present study, although significantly higher HSI scores were observed in the MHO group compared to the MHNO group (Table 2), no significant difference in the risk of MASLD between these groups was noted (Table 4). This finding is not consistent with that of Chen et al. (2021) [17], who analyzed data from 1651 community-based participants. In the present study, the BMI and WC were similar between the MHO and MAO groups (BMI: 28.78 ± 1.16 vs. 29.37 ± 1.83 kg/m^2^; WC: 92.65 ± 7.41 vs. 96.47 ± 5.04 cm), and MASLD was assessed using the HSI. However, Chen et al. reported significantly higher BMI and WC in the MAO group than in the MHO group (BMI: 27.4 ± 2.8 vs. 28.1 ± 2.8 kg/m^2^, *p* < 0.05; WC: 89.2 ± 6.8 vs. 91.8 ± 7.9 cm, *p* < 0.05), and they assessed MASLD through abdominal ultrasonography. Furthermore, our study, which focused on RTRs, with a mean age of 48.09 ± 12.77 years, of whom 58.39% were men, reported an overall MASLD prevalence of 25.55%. This prevalence is substantially lower than the 46.6% observed by Chen et al., whose cohort had an older mean age of 62.9 ± 17.6 years and included 40.1% men. These methodological differences, along with the small sample size and the unique characteristics of our study population, likely account for the differences in findings between our study and those of previous studies.

In the present study, 54.55% of the participants in the MHO group (*n* = 6) were classified as having MASLD. This finding is similar to those of Chen et al., who reported a prevalence of 62.8% [17]. Although the numbers of participants with MHO and those with MASLD in the present study are relatively small, it is speculated that a high proportion of RTRs with MHO may have undiagnosed MASLD. Long-term studies have reported that MHO is not a stable condition and tends to worsen over time. For example, a study involving over 90,000 participants from the Nurses’ Health Study, spanning three decades, revealed that individuals initially classified as metabolically healthy frequently became unhealthy, increasing their risk of cardiovascular disease [18]. Although additional studies are necessary to fully understand the long-term outcomes for RTRs across different obesity subtypes, our results suggest that health-care providers should be vigilant against the risks associated with MHO and MANO in this population. These patients should be regularly monitored for the early onset of metabolic complications.

RTRs who were nonobese but had metabolic abnormalities demonstrated a marginally significant increase in the risk of MASLD compared with those in the MHNO group, with an OR of 2.74 (*p* = 0.056). This result may be attributed to the relatively small sample size in our study. Epidemiological studies have indicated that approximately 3% to 30% of lean or nonobese individuals diagnosed with MASLD experience considerable variability, likely due to differences in patient characteristics, diagnostic methods, or BMI cutoff values for obesity [19]. Kuchay et al. (2021) reviewed studies on lean and nonobese MASLD and uncovered the following clues that may elucidate the underlying mechanisms [20]: (1) lean/nonobese individuals with MASLD tend to have higher amounts of visceral adipose tissue than healthy controls [21] and (2) decreased skeletal muscle mass and impaired muscle function are often exhibited by them, compared to obese individuals with MASLD [22]. On the basis of these findings, we suggest that future studies on the relationship between obesity, metabolic health, and MASLD in RTRs should include a detailed analysis of body composition.

To the best of our knowledge, this is the first study to compare obesity and metabolic health status among RTRs, particularly in the Asian context, and with a relatively large sample size for RTR-related research. However, this study has some limitations. First, causal inference were precluded by the cross-sectional design of the study. Second, because of funding constraints, we used noninvasive scores instead of abdominal ultrasonography or liver biopsy to assess MASLD. Although ultrasonography [23] and liver biopsy [24] are considered the gold standards for diagnosis, they are either too costly for large-scale screening or invasive and less acceptable to patients. However, the HSI, which includes variables such as sex, liver function indicators (ALT and AST), BMI, and diabetic status, is a simple, noninvasive, and validated screening tool for MASLD, with a sensitivity of 89.55%, a negative predictive value of 90.91%, a specificity of 95.24%, and a positive predictive value of 94.49%. Moreover, it was reported that the area under the receiver operating characteristic curve for the HSI was 0.979 (95% CI, 0.962–0.997) for HSI scores above 36 [16]. Third, the definition of metabolic health varies, and different criteria are used across studies [5], making comparisons between studies challenging. There is also considerable debate about the applicability of international obesity criteria to Asian populations, who exhibit different associations between BMI, body fat percentage, and health risks compared to European populations [25]. In this study, obesity was defined as a BMI ≥ 27 kg/m^2^ [11], rather than the WHO’s standard of ≥30 kg/m^2^, and metabolic health was defined in accordance with the criteria for metabolic syndrome established in Taiwan [15]. Using this definition, it was found that the prevalence of MHO was 8.03%, representing 40.74% of all RTRs with obesity, whereas the prevalence of MANO was 24.09%, accounting for 30.00% of RTRs without obesity. Despite the relatively small sample sizes for MHO and MANO in this study, these figures are consistent with those of previous studies, which have reported global prevalence rates of MHO ranging from approximately 10% to 30% [5] and MANO at 26.78% [26]. Moreover, this study is constrained by a small sample size and its single-center design in Asia, limiting its generalizability to the broader population of RTRs. Consequently, the findings may not be applicable to patients from other ethnic backgrounds. Further research is necessary to determine whether our observations can be extrapolated. Finally, future research should identify potential confounding factors that were not explored in this study, including the underlying kidney condition that led to end-stage kidney disease as well as body composition and exercise habits—key components of a healthy lifestyle that can improve physical health—that may affect the development of MASLD in RTRs. Furthermore, other indicators that can assess liver health in RTRs, such as the Fibrosis-4 (FIB-4) index [27], should also be considered.

## 5. Conclusions

This study elucidates the complex relationship among metabolic health, obesity, and liver disease, particularly in RTRs. Our findings indicate that RTRs with both metabolic abnormalities and obesity have a significantly higher prevalence of MASLD, as reflected by their elevated HSI scores. Moreover, even RTRs who are not obese but have metabolic abnormalities have an increased risk of MASLD. These results indicate the importance of comprehensive screening for both obesity and metabolic health to improve long-term health outcomes and prognosis in RTRs. Future studies should validate these findings across diverse RTR populations and clinical settings to enhance their generalizability and practical application.

## Figures and Tables

**Table 1:** Characteristics of 137 RTRs classified by the presence of MASLD ^1^.

	All (*n* = 137)	HSI < 36 (*n* = 102)	HSI > 36 (*n* = 35)
**Demographics**			
Male/female	80/57	60/42	20/15
Age, year	49.20 ± 12.46	48.87 ± 12.52	50.17 ± 12.38
Height, cm	161.91 ± 8.51	161.65 ± 8.22	162.67 ± 9.39
Body weight, kg	62.79 ± 12.68	59.21 ± 10.73	73.23 ± 12.27 *
Body mass index, kg/m^2^	23.83 ± 3.64	22.56 ± 3.01	27.52 ± 2.70 *
WC, cm	83.72 ± 9.53	80.95 ± 8.67	92.04 ± 6.84 *
Renal transplant time, year	8.34 ± 7.01	8.03 ± 7.26	9.25 ± 6.21
Tacrolimus/cyclosporine used	89/42 ^$^	66/30	23/12
Deceased/living donors	96/41	71/31	25/10
**Laboratory**			
Albumin, g/dL	4.32 ± 0.31	4.35 ± 0.29	4.21 ± 0.33 *
Blood urea nitrogen, mg/dL	24.07 ± 10.34	24.14 ± 10.16	23.85 ± 11.02
Creatinine, mg/dL	1.43 ± 0.70	1.41 ± 0.71	1.46 ± 0.66
AC-sugar, mg/dL	99.94 ± 31.79	94.98 ± 17.31	114.26 ± 53.37 *
HbA1c, %	6.04 ± 0.84	5.90 ± 0.77	6.45 ± 0.90 *
Uric acid, mg/dL	6.11 ± 1.28	6.10 ± 1.16	6.16 ± 1.60
Total cholesterol, mg/dL	207.4 ± 46.27	204.19 ± 46.30	216.66 ± 45.57
Triglycerides, mg/dL	162.49 ± 127.99	147.88 ± 122.05	203.83 ± 136.99 *
HDL-C, mg/dL	53.78 ± 17.10	54.98 ± 17.44	50.46 ± 15.89
LDL-C, mg/dL	119.96 ± 37.57	117.69 ± 37.57	126.43 ± 37.35
AST, U/L	25.21 ± 7.84	24.32 ± 6.82	27.80 ± 9.93
ALT, U/L	23.60 ± 17.87	18.18 ± 8.75	39.40 ± 26.53 *
Insulin, mU/L	14.18 ± 50.77	7.70 ± 11.07	31.40 ± 94.21
hs-CRP, mg/dL	6.04 ± 14.61	4.67 ± 12.56	9.83 ± 18.91 *
Dietary intake			
Energy, kcal/day	1810.91 ± 399.53	1821.48 ± 390.94	1780.12 ± 428.00
Carbohydrate, g/day	198.18 ± 55.84	200.49 ± 55.19	191.45 ± 57.98
Carbohydrate, % energy	43.60 ± 7.31	43.87 ± 7.29	42.81 ± 7.42
Protein, g/day	68.02 ± 14.73	67.44 ± 15.02	69.72 ± 13.91
Protein, % energy	15.23 ± 2.56	14.94 ± 2.36	16.06 ± 2.95
Fat, g/day	82.33 ± 21.64	82.98 ± 21.84	80.44 ± 21.22
Fat, % energy	40.99 ± 6.23	41.08 ± 6.44	40.75 ± 5.66
**Others**			
eGFR, mL/min/1.73 m^2^	55.48 ± 20.40	56.52 ± 21.38	52.45 ± 17.15
SBP, mmHg	133.94 ± 16.38	133.28 ± 15.30	135.88 ± 19.31
DBP, mmHg	77.72 ± 12.05	77.47 ± 11.87	78.47 ± 12.68
CCI	2.01 ± 1.17	2.03 ± 1.19	1.94 ± 1.11
HOMA-IR	3.37 ± 13.56	1.62 ± 1.18	8.34 ± 26.18 *
HSI	32.39 ± 6.03	29.67 ± 3.66	40.33 ± 4.31 *

Abbreviations: RTRs, renal transplant recipients; MASLD, metabolic-associated steatotic liver disease; HSI, hepatic steatosis index; WC, waist circumference; AC-sugar, preprandial blood glucose; HbAlC, glycated hemoglobin A1c; HDL-C, high-density lipoprotein cholesterol; LDL-C, low-density lipoprotein cholesterol; AST, aspartate aminotransferase; ALT, alanine aminotransferase; hs-CRP, high-sensitivity C-reactive protein; eGFR, estimated glomerular filtration rate; SBP, systolic blood pressure; DBP, diastolic blood pressure; CCI, Charlson comorbidity index; HOMA-IR, homeostasis model assessment-insulin resistance. ^1^ Values are presented as the mean ± standard deviation or number, as appropriate. Statistical analyses were conducted using Student’s *t*-test, Wilcoxon rank sum test, and chi-square test as appropriate. * *p* < 0.05. ^$^ No records of six patients.

**Table 2:** Characteristics of 137 RTRs according to obesity phenotypes ^1^.

	MHNO (*n* = 77)	MHO (*n* = 11)	MANO (*n* = 33)	MAO (*n* = 16)	*p* for Trend
**Demographics**					
Male/female	45/32	8/3	15/18	12/6	0.173
Age, year	48.09 ± 12.77	51.18 ± 7.24	53.21 ± 11.27	44.94 ± 14.51	0.102
Height, cm	161.39 ± 8.17	162.55 ± 8.70	161.56 ± 8.73	164.69 ± 9.77	0.554
Body weight, kg	57.22 ± 9.93 ^a^	76.26 ± 8.80 ^b^	62.90 ± 8.34 ^a^	80.13 ± 12.07 ^b^	<0.0001
Body mass index, kg/m^2^	21.87 ± 2.62 ^a^	28.78 ± 1.16 ^c^	24.07 ± 2.21 ^b^	29.37 ± 1.83 ^c^	<0.0001
WC, cm	79.09 ± 7.77 ^a^	92.65 ± 7.41 ^b^	85.64 ± 7.16 ^a^	96.47 ± 5.04 ^b^	<0.0001
Renal transplant time, year	7.43 ± 6.84	12.97 ± 5.36	9.38 ± 8.17	7.42 ± 4.81	0.066
Tacrolimus/cyclosporine used ^$^	51/21	6/5	18/14	14/2	0.113
Deceased/living donors	53/24	8/3	25/8	10/6	0.797
**Laboratory**					
Albumin, g/dL	4.34 ± 0.30 ^a^	4.03 ± 0.34 ^b^	4.34 ± 0.21 ^a^	4.36 ± 0.40 ^c^	0.016
Blood urea nitrogen, mg/dL	23.54 ± 9.28	24.48 ± 7.23	26.15 ± 13.86	21.90 ± 8.37	0.532
Creatinine, mg/dL	1.35 ± 0.57	1.39 ± 0.54	1.57 ± 1.00	1.49 ± 0.58	0.482
AC-sugar, mg/dL	89.71 ± 11.85 ^a^	93.73 ± 11.45 ^a^	114.06 ± 22.97 ^a^	125.19 ± 74.42 ^b^	<0.0001
HbA1c, %	5.72 ± 0.50 ^a^	6.07 ± 0.46 ^b^	6.60 ± 1.07 ^b^	6.43 ± 1.05 ^b^	<0.0001
Uric acid, mg/dL	6.25 ± 1.26	6.34 ± 1.20	5.61 ± 1.30	6.36 ± 1.24	0.074
Total cholesterol, mg/dL	205.62 ± 46.61	201.55 ± 43.34	214.91 ± 45.57	204.38 ± 50.44	0.751
Triglycerides, mg/dL	110.92 ± 45.99	134.45 ± 68.69	241.52 ± 176.38	257.31 ± 168.58	<0.0001
HDL-C, mg/dL	59.79 ± 16.93 ^a^	52.45 ± 14.11 ^b^	44.75 ± 13.93 ^b^	45.31 ± 14.73 ^b^	<0.0001
LDL-C, mg/dL	120.11 ± 38.97	119.82 ± 32.17	122.33 ± 36.13	114.44 ± 40.00	0.925
AST, U/L	23.57 ± 6.72	26.64 ± 8.12	27.30 ± 8.71	27.81 ± 9.52	0.048
ALT, U/L	19.65 ± 12.72	27.36 ± 20.03	27.24 ± 23.42	32.50 ± 20.79	0.021
Insulin, mU/L	5.96 ± 2.93 ^a^	9.83 ± 4.37 ^a^	11.24 ± 17.90 ^a^	58.16 ± 136.69 ^b^	0.002
hs-CRP, mg/dL	3.42 ± 4.18	6.40 ± 7.96	12.21 ± 27.52	5.38 ± 7.64	0.042
**Others**					
Energy, kcal/day	1849.23 ± 371.76	1955.94 ± 382.84	1733.8 ± 385.04	1685.81 ± 529.46	0.175
eGFR, mL/min/1.73 m^2^	57.75 ± 20.41	57.09 ± 20.80	50.03 ± 21.49	54.69 ± 17.19	0.336
SBP, mmHg	130.13 ± 15.53	133.91 ± 13.14	138.43 ± 16.09	143.03 ± 18.34	0.008
DBP, mmHg	75.94 ± 11.83	76.29 ± 6.98	80.64 ± 13.35	81.28 ± 11.99	0.160
CCI	1.87 ± 1.18	2.00 ± 1.55	2.39 ± 0.97	1.88 ± 1.09	0.180
HOMA-IR	1.41 ± 1.11 ^a^	2.37 ± 1.14 ^a^	2.38 ± 1.34 ^a^	15.88 ± 39.03 ^b^	0.003
HSI	29.50 ± 4.55 ^a^	38.08 ± 5.44 ^b^	33.61 ± 5.23 ^a^	39.86 ± 4.13 ^b^	<0.0001

Abbreviations: RTRs, renal transplant recipients; MHNO, metabolically healthy nonobese; MHO, metabolically healthy obese; MANO, metabolically abnormal nonobese; MAO, metabolically abnormal obese; WC, waist circumference; AC-sugar, preprandial blood glucose; HbAlC, glycated hemoglobin A1c; HDL-C, high-density lipoprotein cholesterol; LDL-C, low-density lipoprotein cholesterol; AST, aspartate aminotransferase; ALT, alanine aminotransferase; hs-CRP, high-sensitivity C-reactive protein; eGFR, estimated glomerular filtration rate; SBP, systolic blood pressure; DBP, diastolic blood pressure; CCI, Charlson comorbidity index; HOMA-IR, homeostasis model assessment-insulin resistance; HSI, hepatic steatosis index. ^1^ Values are presented as the mean ± standard deviation or number, as appropriate. Statistical analyses were conducted using analysis of variance (ANOVA), and *p* values refer to the overall ANOVA among the four groups. Post hoc analyses were performed using Scheffe’s adjustment for multiple comparisons. Category means not accompanied by the same letter symbol are significantly different from each other. ^$^ No records of six patients.

**Table 3:** Multiple linear regression analysis for MASLD by using the HSI as a dependent variable by obesity phenotypes in 137 RTRs ^1^.

	Crude Model			Model 1			Model 2		
	β	95% CI	*p*	β	95% CI	*p*	β	95% CI	*p*
**HSI**									
MHNO	1			1			1		
MHO	8.58	4.89–12.27	<0.0001	8.43	4.71–12.15	<0.0001	8.01	4.11–11.91	<0.0001
MANO	4.11	1.73–6.50	<0.0001	3.88	1.45–6.31	0.0002	4.38	1.81–6.94	<0.0001
MAO	10.36	7.21–13.51	<0.0001	10.50	7.33–13.68	<0.0001	10.96	7.63–14.29	<0.0001

Abbreviations: RTRs, renal transplant recipients; MASLD, metabolic-associated steatotic liver disease; HSI, hepatic steatosis index; CI, confidence interval; MHNO, metabolically healthy nonobese; MHO, metabolically healthy obese; MANO, metabolically abnormal nonobese; MAO, metabolically abnormal obese. ^1^ β refers to the regression coefficient determined to assess the association between obesity phenotypes as a dependent variable and the HSI as an independent variable. Model 1: adjustment for age and sex. Model 2: Model 1 + adjustment for total energy intake, comorbidity, kidney transplant date, donor source, and immunosuppressant use.

**Table 4:** Multivariate logistic analysis for MASLD Using HSI (>36) according to obese phenotypes in 252 RTRs ^1^.

	Crude Model			Model 1			Model 2		
	OR	95% CI	*p*	OR	95% CI	*p*	OR	95% CI	*p*
**HSI**									
MHNO	1			1			1		
MHO	11.11	2.72–45.39	0.304	11.31	2.68–47.80	0.305	7.91	1.74–35.91	0.697
MANO	3.13	1.05–9.75	0.058	2.61	0.86–7.88	0.027	2.74	0.88–8.52	0.056
MAO	54.42	11.26–264.01	0.0002	66.34	12.69–346.75	0.0001	74.59	13.29–418.68	0.0001

Abbreviations: RTRs, renal transplant recipients; MASLD, metabolic-associated steatotic liver disease; HSI, hepatic steatosis index; OR, odds ratio; CI, confidence interval; MHNO, metabolically healthy nonobese; MHO, metabolically healthy obese; MANO, metabolically abnormal nonobese; MAO, metabolically abnormal obese. ^1^ Statistical analyses were conducted using logistic regression analysis. Model 1: adjustment for age and sex. Model 2: model 1 + adjustment for total energy intake, comorbidity, kidney transplant date, donor source, and immunosuppressant use.

## Data Availability

The data supporting the findings of this study are available from the corresponding author upon reasonable request. Due to ethical restrictions, the data are not publicly accessible.
